# Dosimetric impact of the positioning variation of tumor treating field electrodes in the PriCoTTF‐phase I/II trial

**DOI:** 10.1002/acm2.13144

**Published:** 2021-01-03

**Authors:** Youness Nour, Christoph Pöttgen, Sied Kebir, Lazaros Lazaridis, Lutz Lüdemann, Maja Guberina, Thomas Gauler, Björn Scheffler, Ramazan Jabbarli, Daniela Pierscianek, Ulrich Sure, Teresa Schmidt, Christoph Oster, Peter Hau, Martin Glas, Wolfgang Lübcke, Martin Stuschke, Nika Guberina

**Affiliations:** ^1^ Department of Radiotherapy West German Cancer Center University Hospital Essen University of Duisburg Essen Germany; ^2^ Division of Clinical Neurooncology Department of Neurology and West German Cancer Center University Hospital Essen University of Duisburg Essen Germany; ^3^ DKFZ‐Division Translational Neurooncology at the West German Cancer Centre (WTZ) German Cancer Consortium (DKTK) Partner Site University Hospital Essen University of Duisburg Essen Germany; ^4^ German Cancer Consortium (DKTK) Partner Site University Hospital Essen Germany; ^5^ Department of Neurosurgery University Hospital Essen University of Duisburg Essen Germany; ^6^ Department of Neurology and Wilhelm Sander‐NeuroOncology Unit Regensburg University Hospital Germany

**Keywords:** dosimetry, glioblastoma, non‐coplanar IMRT, tumor treating fields

## Abstract

**Purpose:**

The aim of the present study based on the PriCoTTF‐phase I/II trial is the quantification of skin‐normal tissue complication probabilities of patients with newly diagnosed glioblastoma multiforme treated with Tumor Treating Field (TTField) electrodes, concurrent radiotherapy, and temozolomide. Furthermore, the skin‐sparing effect by the clinically applied strategy of repetitive transducer array fixation around their center position shall be examined.

**Material and Methods:**

Low‐dose cone‐beam computed tomography (CBCT) scans of all fractions of the first seven patients of the PriCoTTF‐phase I/II trial, used for image guidance, were applied for the dosimetric analysis, for precise TTField transducer array positioning and contour delineation. Within this trial, array positioning was varied from fixation‐to‐fixation period with a standard deviation of 1.1 cm in the direction of the largest variation of positioning and 0.7 cm in the perpendicular direction. Physical TTField electrode composition was examined and a respective Hounsfield Unit attributed to the TTField electrodes. Dose distributions in the planning CT with TTField electrodes in place, as derived from prefraction CBCTs, were calculated and accumulated with the algorithm Acuros XB. Dose‐volume histograms were obtained for the first and second 2 mm scalp layer with and without migrating electrodes and compared with those with fixed electrodes in an average position. Skin toxicity was quantified according to Lyman's model. Minimum doses in hot‐spots of 0.05 cm^2^ and 25 cm^2^ (ΔD_0.05cm_^2^, ΔD_25cm_^2^) size in the superficial skin layers were analyzed.

**Results:**

Normal tissue complication probabilities (NTCPs) for skin necrosis ranged from 0.005% to 1.474% (median 0.111%) for the different patients without electrodes. NTCP logarithms were significantly dependent on patient (*P* < 0.0001) and scenario (*P* < 0.0001) as classification variables. Fixed positioning of TTField arrays increased skin‐NTCP by a factor of 5.50 (95%, CI: 3.66–8.27). The variation of array positioning increased skin‐NTCP by a factor of only 3.54 (95%, CI: 2.36–5.32) (*P* < 0.0001, comparison to irradiation without electrodes; *P* = 0.036, comparison to irradiation with fixed electrodes). NTCP showed a significant rank correlation with D25cm^2^ over all patients and scenarios (r_s_ = 0.76; *P* < 0.0001).

**Conclusion:**

Skin‐NTCP calculation uncovers significant interpatient heterogeneity and may be used to stratify patients into high‐ and low‐risk groups of skin toxicity. Array position variation may mitigate about one‐third of the increase in surface dose and skin‐NTCP by the TTField electrodes.

## 1 INTRODUCTION

Concurrent tumor‐treating field (TTField)‐ and radiotherapy treatment is under dynamic discussion, particularly since preclinical studies suggested that its combination may comprise an enhanced clinical efficacy.^1^, ^2^, ^3^ This is attributed to the ability to serve as a radiosensitizer.^1^ Conversely, an adverse effect of concurrent TTField‐ and radiotherapy treatment reported is TTFields‐related skin toxicity.^4^ Previous studies showed that concurrent TTField‐ and radiotherapy treatment may cause both, an increased buildup effect and an increased back scatter effect on exit dose, leading to increased skin toxicity.^5^ This merits watchfulness for the clinical use of concurrent TTField‐ and radiotherapy treatment.^5^ Some authors suggest that caution should be exercised when considering therapeutic radiation with TTField arrays in place, as their results highlight potentially prohibitive skin toxicity.^5^ Phantom studies implied that wearing transducer arrays during radiotherapy should not lead to a clinically significant underdosage of the target volume due to the attenuation of the treatment beams.^6^, ^7^ Yet, increased skin doses were noticed.^6^, ^7^ Without radiotherapy, Stupp et al. report only a mild to moderate skin toxicity from transducer arrays.^8^

As a new concept, simultaneous radiotherapy with TTFields is conducted within the multicenter PriCoTTF‐phase I/II trial (European database on medical devices (Eudamed) CIV 18–08‐025247). The clinical I/II trial examines the efficacy of TTField electrodes with concurrent radiotherapy and temozolomide in patients with newly diagnosed glioblastoma multiforme (GBM). Skin reaction of grade III–IV is the primary endpoint of the PriCoTTF‐phase I/II trial. The secondary endpoint of the trial is the dosimetric skin exposure. A previous study from our group showed that in the first seven patients included in the PriCoTTF‐phase I/II trial dose deviations in the CTV due to transducer arrays were not clinically significant confirming feasibility of combined adjuvant radiochemotherapy and concurrent TTFields from a dosimetric point of view.^9^ The dose buildup in the skin resulted in a dose increase of below 8.5% outside the “hottest” 1 cm^2^ with TTFields which moved around their center position.^9^ The present dosimetric analysis of the dose distributions in the outer skin layer, calculated by a clinical Acuros XB algorithm (Acuros XB, Eclipse version 15.5, Varian Medical Systems, Palo Alto, CA, USA), is anchored in the PriCoTTF‐phase I/II trial. The suitability of the Lyman NTCP Model^10^, ^11^ based on the clinical tolerance table by Emami et al.^12^ is analyzed to detect interpatient differences. Furthermore, the effect of TTField electrodes and the mitigation effect of variation of array positions around their center on skin normal tissue toxicities shall be examined.

## 2 METHODS

The present dosimetric analysis is based on the first seven patients of the PriCoTTF‐phase I/II trial. Prior to concurrent TTField radiotherapy patient cases were discussed in an interdisciplinary, neurooncological tumor board. Inclusion criteria were a newly diagnosed, histopathologically confirmed glioblastoma, age ≤70 years and Karnofsky performance status (KPS) ≥ 60% or age ≥70 years and KPS ≥ 50%. Written informed consent was obtained from all patients previous to concurrent TTField radiotherapy. Four transducer arrays, each consisting of nine TTField electrodes on an adhesive tape, were fixed on the skin of the head in an anterior, posterior, left lateral, and right lateral position. The arrays were changed every 3–4 days. Prior to fixation of new arrays, the skin was allowed to recover for 4–6 hrs without electrodes. At the days of array change, arrays were relocated after the daily radiation fraction and therefore patients were irradiated without the arrays. In addition, patients got the instruction to vary the position of the transducer arrays by about half an electrode diameter, viz. 1 cm, at each change of the transducer arrays in the plane of the skin. This variation of TTField array position from fixation period to fixation period resulted in 95% confidence ellipses with a mean half‐length of the major axis of about 2.7 cm and of the minor axis of about 1.8 cm.^9^

### 2.1 Radiotherapy planning and treatment

The planning CT scan was performed on a multislice‐detector computed tomography scanner (Siemens Healthineers, Erlangen, Germany) with low osmolar, nonionic contrast medium without TTField arrays in place. The planning CT scan was rigidly fused with an up‐to‐date, postoperative MRI scan using a three‐dimensional radiotherapy treatment planning system (Eclipse, Varian Medical Systems, Palo Alto, CA, USA). Radiotherapy planning was based on 1 mm, contrast‐enhanced postoperative, 3D volumetric interpolated breath‐hold examination (VIBE) as well as on fluid‐attenuated inversion recovery (FLAIR) sequences. With regard to anatomical boundaries clinical target volume (CTV) was delineated with a 2 cm margin around the gross tumor volume (GTV) including suspicious FLAIR hyperintensities. Additionally, 2–5 mm were expanded around the CTV for definition of the planning target volume (PTV). After delineation of organs at risk the final radiotherapy plan stated that the maximum dose at the brainstem must not surpass 54 Gy, as well as 55 Gy at the chiasm and the optic nerves using a normofractionation scheme. Using a hypofractionation scheme the maximum dose must not top 40 Gy at the brainstem, chiasm, and optic nerve, respectively.

Radiotherapy was conducted normofractionated daily with 2 Gy/F ad 60 Gy in arm A or hypofractionated with 2.67 Gy/F ad 40.05 Gy in arm B, viz. in elderly or in patients with a reduced general condition, following NCCN guidelines.^13^ The dose coverage of the PTV was determined ≥90% of the PTV volume and the D98 > 95%. Noncoplanar, intensity‐modulated radiotherapy (IMRT) fields with 6 MV photons were applied at Varian TrueBeam linear accelerator system (LINAC, Varian Medical Systems, Palo Alto, CA, US). Contrary to the planning CT, radiotherapy treatment was performed with TTField transducer arrays in place and switched off. After radiotherapy treatment TTField transducer arrays were turned on again.

### 2.2 Dose accumulation

Low‐dose Cone‐Beam Computed Tomography (CBCT) scans of all fractions used for image guidance, were applied for the dosimetric analysis as well as for the precise TTField positioning and contour delineation. The FOV of the CBCT covered the skull above the orbit excluding the eye lens. The physical TTField electrode composition was examined and a respective Hounsfield Unit of 3832 HU was attributed to the TTField electrodes as described in Ref. [9]. Dose distributions with TTField electrodes contoured and overwritten with a density characterized by 3832 HU in the planning CT at all array positions observed on all prefraction CBCT's were calculated and accumulated with the Boltzmann equation solver implemented in the algorithm Acuros XB (Acuros XB, Eclipse version 15.5, Varian Medical Systems, Palo Alto, CA, USA) as described in Ref. [9]. Although the Acuros XB algorithm is known to comprise some uncertainties at the interface of water and high Z‐material, our previous work demonstrated that Acuros XB exhibits an excellent correlation with the Monte Carlo Simulation algorithm implemented in Prosoma version 4.2. (MedCom, Darmstadt, Germany) (based on the VMC++ and XVMC‐ code)^9^: (a) for the superficial 2 mm layer without TTField electrodes the Spearman correlation coefficient was 0.937**, *P* = 0.002; (b) for the superficial 4 mm layer without TTField electrodes 0.929**, *P* = 0.003; (c) for the superficial 2 mm layer with TTField electrodes 1.000**, *P* < 0.0001; and (d) for the superficial 4 mm layer with TTField electrodes 0.929**, *p* = 0.003 (Suppl. Figure S1). Therefore, we surrendered the recalculation of the dose distributions for the superficial layers with the Monte Carlo Simulation algorithm and focused on the clinical Acuros XB dose calculation. The dose calculation grid was set to 1.5 mm resolution. The accumulated dose distribution with the observed varying electrode positions was renormalized by the factor N = 60 Gy / (number of fractions with CBCT's *prescribed dose per fraction) for all patients. This accumulated dose distribution was compared with the dose distribution without TTField arrays. In a next section, an attempt was made to define further possible scenarios for the positioning of the TTFields. The dose distribution was recalculated for a virtually fixed TTField array position nearest to the average position on the scalp over the whole series. The following five scenarios of TTField array positioning were defined and compared with each other:

Scenario 1: Dose distributions were calculated without TTField electrodes.

Scenario 2: Dose distributions were calculated with observed varying electrode positions.

Scenario 3: Dose distributions were recalculated with virtually fixed TTField electrodes nearest average position.

Scenario 4: Dose distributions were calculated as ^2^/_5_ of fractions given under scenario 1 and ^3^/_5_ under scenario 3.

Scenario 5: Dose distributions were calculated as ^2^/_5_ of fractions given under scenario 1 and ^3^/_5_ under scenario 2.

The area of skin tolerance for 60 Gy with normofractionation lies below 30 cm^2^.^12^ Therefore, from the individual plans for scenarios 1–3, Δ D_25cm_^2^ was determined as the lowest dose in the 25 cm^2^ at the highest dose in the superficial 2 mm layer of skin.

### 2.3 Normal Tissue Complication Probability (NTCP) for skin

Nowadays data on skin toxicities by treatment with megavoltage beams from linear accelerators are scant. Emami et al. estimated skin tolerance data for homogeneously irradiated skin independence on the skin area.^12^ Endpoint was skin necrosis and ulceration. From these data, Burman et al. estimated the parameters for the Lyman model.^10^, ^11^ The Lyman model and the Kutcher Burman volume reduction algorithm allows to determine the isoeffective, homogeneously exposed partial volume to a given references dose for an inhomogeneously irradiated structure, here 70 Gy with a conventional fractionation that would result in the same NTCP.^14^ The NTCP is obtained from a normal distribution with an organ and endpoint specific slope‐determining variance parameter. We calculated the NTCPs for the DVH's in the outer 2 mm layer 0–2 mm below the surface and the next deeper shell from 2 to 4 mm depth. In addition, the *g*EUD (generalized Equivalent Uniform Dose) is calculated as the isotoxic uniform dose to be administered to the organ at risk, here 100 cm^2^ of skin, which results in the same NTCP as the actually inhomogeneously applied dose.^11^, ^15^, ^16^, ^17^

### 2.4 Statistical analysis

For statistical analyses SAS (version 14.1, SAS Institute, Cary, NC, USA) was applied. The Kolmogorov–Smirnov test from the procedure Univariate was used to determine normality, the procedure GLM was used to calculate the analyses of variance and the procedure CORR to calculate rank correlations.

## 3 RESULTS

**FIG. 1:**
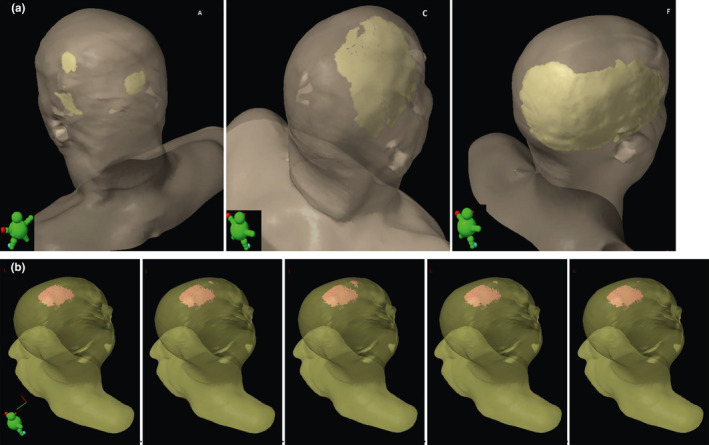
(a) Model view showing the V40 isodose distribution on the outer skin layer 0‐2mm for patients A (left), C (middle), and patient F (right). (b) Model view delineating the V40 isodose distribution on the outer skin layer 0‐2 mm for patient G. Scenarios 1 to 5 are shown from left to right

**FIG. 2:**
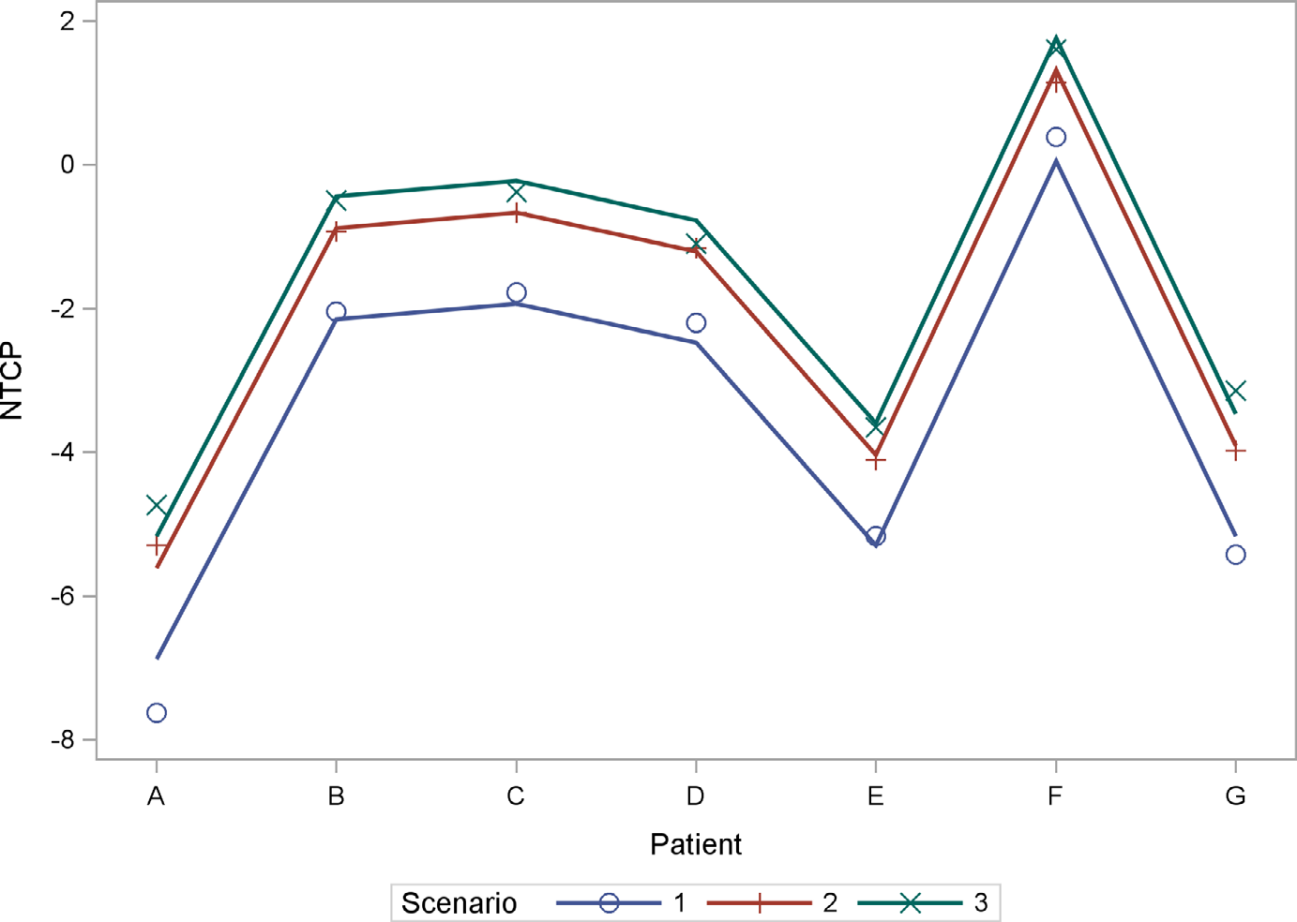
Highlighting interaction plot for normal tissue complication probability (NTCP): dependence of the log_NTCP_ on patients and scenarios 1‐3 and the values predicted by the analysis of variance

**FIG. 3:**
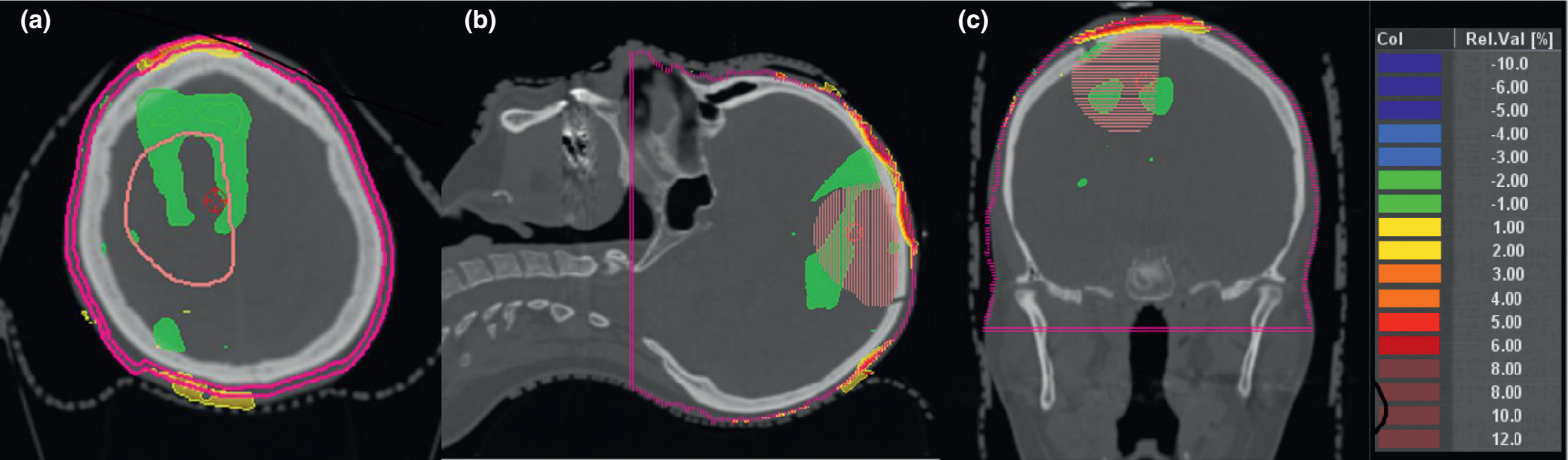
(a–c) Depiction of dose buildup in the superficial scalp layer (2 mm shell contour): Highlighting dose difference plots of accumulated dose distributions without and with TTField electrodes which were fixed at varying positions around a center position during the course of the treatment. Delineated clinical target volume (right frontoparietal), (a) right: axial computed tomography; (b) center: sagittal reconstruction; (c) left: coronar reconstruction. Dose differences are expressed as percentages of the prescribed dose

The minimum doses to the highest exposed 0.05 cm^2^ and 25 cm^2^ of skin are shown in Table 1 for the seven patients and scenarios 1–5. Analysis of variance showed a significant patient and scenario dependent effect on the D25 cm^2^ (*P* < 0.0001, ANOVA F‐test). Scenarios 2 and 3 showed higher D25 cm^2^ values than scenario 1. The difference between scenarios 3 and 2 with 1.07 Gy (SD 0.45 Gy, *P* = 0.03 F‐test) was less than the difference between scenarios 2 and 1 with 3.49 Gy (SD 0.45 Gy, *P* < 0.0001 F‐test) or between 3 and 1 with 4.56 Gy (SD 0.45 Gy, *P* < 0.0001 F‐test). Normal tissue toxicity for the superficial 0‐2 mm skin layer according to the Lyman model are given in Table 2,^10^ the *g*EUD values according to the Luxton model^18^ in Table 3. While *g*EUD and NTCP showed a perfect rank correlation (r_s_ = 1.00) according to their functional relation, D25 cm^2^ and NTCP showed a moderate to good rank correlation over all patients and the scenarios 1–3 (r_s_ = 0.76; *P* < 0.0001, t‐test). Figure 1(a) visualizes the skin areas receiving more than 40 Gy in total dose for patients A, C, and F, with the lowest, second highest, and highest skin complication probability. Figure 1(b) visualizes the V40 isodose distribution on the outer skin layer 0–2 mm for patient G. NTCP for skin differed significantly between patients and scenarios as classification variables (*P* < 0.0001, for each factor). Relevant NTCPs above 1% appeared only in patient F. Analysis of variance was not performed on NTCPs but the logarithms of the NTCPs (log_NTCP_), as the former were not compatible with a normal distribution (*P* < 0.01, Kolmogorov–Smirnov test), while the latter were normally distributed (*P* > 0.15, Kolmogorov–Smirnov test). Figure 2 shows the dependence of the log_NTCP_ on patients and scenarios 1–3 and the values predicted by the analysis of variance. Scenario 3 with fixed observed positioning of TTField arrays nearest to the average position increased the skin NTCP by a factor of 5.50 (95%CI: 3.66–8.27). Fixed electrode position may lead to a clinically important increase in NTCP from 1.5% to 5.0% as shown for patient F, while for the other patients the absolute increase in NTCP was below 0.6%. Varying array positioning from fixation period to fixation period increased the NTCP lesser by a factor of only 3.54 (95% CI: 2.36–5.32) (*P* < 0.0001 compared to the irradiation without electrodes; *P* = 0.036 comparison to fixed electrodes, F‐test). Combining the variation of array position with irradiation without electrodes 2 days per week, the days of array change, reduced the increase in skin NTCP in comparison to irradiation without electrodes further to a factor 1.88 (95% CI: 1.24–2.83) (*P* = 0.0049, F‐test, scenario 5 vs. scenario 1). Table 4 shows together with the data in Table 3 the increase in *g*EUD with depth below surface for the layers of 0–2 mm and 2–4 mm below surface. The *g*EUD increased by 3.21 Gy (95% CI: 2.87 Gy–3.54 Gy) from layer 0–2 mm to layer 2–4 mm and this increase was slightly dependent on the patient and the scenario. The increase in *g*EUD was largest for scenario 1 with 4.02 Gy (95% CI: 3.55 Gy–4.48 Gy) and least for scenario 3 with 2.57 Gy (95% CI: 2.10 Gy–3.03 Gy). Figure 3 highlights the spatial distribution of the dose differences between the scenario 2 and scenario 1 dose distributions.

**TABLE 1:** Minimum doses in the a) 0.05 cm^2^ and b) 25 cm^2^ of skin at highest dose from the dose‐volume histograms for the five different scenarios in the first seven patients A–G of the PriCoTTF‐phase I/II trial [in Gy].

Patient	PTV Volume in cm^3^	Scenario 1	Scenario 2	Scenario 3	Scenario 4	Scenario 5
a) D0.05cm^2^ for the skin layer 0‐2 mm below surface [Gy]						
A	168.19	45.3	48.9	52.3	47.8	47.0
B	242.66	62.4	64.7	67.9	64.9	63.6
C	361.77	58.2	62.5	64.8	61.5	60.2
D	365.68	62.7	66.0	65.4	63.5	64.3
E	190.45	60.7	60.4	62.2	61.5	60.6
F	556.36	61.8	63.2	66.3	63.5	62.0
G	107.51	53.1	58.1	61.0	57.2	55.6
b) D25cm^2^ for the skin layer 0‐2 mm below surface [Gy]						
A	168.19	33.1	37.9	36.8	35.1	35.4
B	242.66	36.8	41.4	43.9	40.7	39.4
C	361.77	46.2	49.5	50.3	48.1	48.0
D	365.68	42.8	46.0	46.7	44.5	44.4
E	190.45	32.6	36.3	36.7	35.3	35.2
F	556.36	52.8	55.3	57.3	55.3	54.6
G	107.51	28.8	31.7	32.2	31.4	31.0

**TABLE 2:** Calculated NTCP (in %) for the 0‐2 mm superficial skin layer calculated according to the Lyman model for the five different scenarios in the first seven patients A–G of the PriCoTTF‐phase I/II trial according to the Lyman model.

NTCP [%] for shell 0‐2 mm						
Patient	PTV Volume in ccm	Scenario 1	Scenario 2	Scenario 3	Scenario 4	Scenario 5
A	168.19	0.0005	0.0050	0.0088	0.0021	0.0016
B	242.66	0.1303	0.3951	0.6070	0.2774	0.2455
C	361.77	0.1697	0.5131	0.6820	0.3174	0.3069
D	365.68	0.1109	0.3146	0.3335	0.1790	0.1878
E	190.45	0.0057	0.0165	0.0258	0.0124	0.0101
F	556.36	1.4744	3.1408	5.0063	2.5758	2.2159
G	107.51	0.0044	0.0188	0.0433	0.0140	0.0097

**TABLE 3:** Calculated *g*EUD (in Gy) of the 0‐2 mm skin layer for the five different scenarios in the first seven patients A–G of the PriCoTTF‐phase I/II trial according to Luxton et al.^18^

*g*EUD for shell 0‐2 mm						
Patient	PTV Volume in ccm	Scenario 1	Scenario 2	Scenario 3	Scenario 4	Scenario 5
A	168.19	32.85	37.30	38.48	35.55	35.03
B	242.66	44.71	47.68	48.93	46.7	46.37
C	361.77	45.39	48.44	49.28	47.07	46.98
D	365.68	44.30	47.05	47.21	45.53	45.66
E	190.45	37.59	39.83	40.84	39.21	38.78
F	556.36	51.71	54.37	56.19	53.64	53.11
G	107.51	37.05	40.12	42.02	39.48	38.70

**TABLE 4:** Calculated *g*EUD (in Gy) of the skin layer in 2‐4 mm depth for the five different scenarios in the first seven patients A–G of the PriCoTTF‐phase I/II trial.

*g*EUD for shell 2‐4 mm						
Patient	PTV Volume in ccm	Scenario 1	Scenario 2	Scenario 3	Scenario 4	Scenario 5
A	168.19	39.98	41.81	41.86	40.97	40.91
B	242.66	51.17	52.25	52.61	51.92	51.80
C	361.77	53.38	54.27	54.54	53.94	53.89
D	365.68	52.41	53.27	53.19	52.79	52.87
E	190.45	46.14	47.11	47.22	46.72	46.74
F	556.36	59.16	59.92	60.20	59.67	59.58
G	107.51	47.61	48.80	49.29	48.52	48.28

## 4 DISCUSSION

The present results based on the PriCoTTF‐phase I/II trial reveal that the geometric variation of the TTField array positioning comprises substantial dosimetric consequences on patient skin dose during concomitant TTField radiochemotherapy treatment. The precise positioning, alignment, and adjustment of TTFields from change‐to‐change is important. Previous authors discussed TTFields’ intensity and anisotropy^19^ pointing out that its distribution may be affected by skull morphology,^20^ tumor location and extent,^21^ local differences in tissue conductivity^20^ and last but not least transducer array design.^21^ The clinical application of TTFields typically demands a circular TTField shift from array to array fixation. Prior to treatment initiation patients are instructed by a medical device expert how to apply TTFields in everyday life using these as a minimum 75% of the time, viz. 18 hrs per day. The precise alignment and geometric variation of the TTFields, which are altered every 3 days, depend on the tumor localization and the clinical device plan provided by the vendor. The dosimetric influence of concomitant radiotherapy on TTField treatment has been described in both, several phantom studies^5^, ^6^, ^7^ and a human phase I/II trial.^9^ Principle component analysis (PCA) of the TTField migration derived from the CBCTs confirmed that the migration in spatial direction with the highest variation of TTFields comprises a standard deviation of 1.0–1.2 cm for the frontal, occipital, left‐ and right‐sided arrays as well as 0.7–0.8 cm for the orthogonal principal component.^9^ This migration leads to a diminution of the dosimetric impact of the TTFields.^9^ Moreover, the results are also plan specific. Likewise, the present is the first study which examines the dependency of dose distribution of concomitant TTField radiotherapy treatment on the TTField positioning variation in the human model of a phase I/II trial.

Skin‐related toxicity is a well‐known adverse event incidence in patients treated with TTFields, which may be enhanced by various factors.^4^, ^5^, ^22^ Therefore, according to the manufacturer the skin is allowed to be uncovered from the arrays for at least 4 hrs from array to array fixation. A prospective, randomly controlled pivotal phase III trial (Trial registration: clinicaltrials.gov Identifier: NCT00916409) examined the efficacy and safety of TTFields, as an adjuvant to the best standard of care in the treatment of patients with newly diagnosed glioblastoma.^22^ In this phase III trial aimed to investigate the effect of TTFields plus maintenance temozolomide vs maintenance temozolomide alone Stupp et al. report that the only adverse event incidence normalized to duration of treatment was a higher incidence of localized skin toxic effects.^22^ TTField treatment was conducted at least 4 weeks from the last day of radiotherapy.^22^ Stupp et al. observed that beneath the transducer arrays on the medical device site patients treated with TTFields plus temozolomide suffered from mild to moderate skin irritation in 52%, and from severe skin reaction grade 3 in 2%.^22^ Likewise, Bokstein et al. examined in a single‐arm trial the safety of concomitant TTFields prior to or at the time of radiotherapy with daily removal of the transducer arrays during radiotherapy delivery.^4^ Although all TTFields‐related skin toxicities were of low severity (CTCAE grade 1–2), these were observed in 80% of patients.^4^ The PriCoTTF‐phase I/II trial is the first which examines the efficacy of Tumor TTField electrodes with concurrent radiotherapy and temozolomide without daily removal of the transducer arrays during radiotherapy. Radiotherapy treatment is performed through the switched‐off TTField transducer arrays. For the first time in men, based on the PriCoTTF‐phase I/II trial, it could be confirmed from a dosimetric point of view that the translation of a combined radiochemo‐TTF‐based therapy into the clinical setting is feasible.^9^

The basal cell layer of the epidermis is the target for radiation‐induced skin toxicity and is located in a depth of 0.07 mm. This depth should be considered for skin dose measurements.^23^ Radiation doses in such superficial target volumes may be measured by film dosimetry.^24^ However, these are impractical in the clinical routine underneath the TTField arrays with changing array positions from fixation period to fixation period. Skin dose depends on field size, beam energy, beam modifying devices, obliquity of the fields, curvature of the patient surface and other factors.^25^ Here, we used the superficial layer of 2 mm thickness from 0 to 2 mm below body surface, that is, at an average depth of 1 mm, to estimate skin toxicity. Layers of a 2 mm thickness can be created with reasonable precision by Boolean operations using clinical planning systems, and are adapted to the resolution of the CT images and the dose calculation grid of the clinical planning systems. A good agreement between film measurements and dose calculations with an anisotropic analytical algorithm was found in adjacent shells of 2 mm thickness centered around a depth of 2, 4, and 6 mm in an head and neck wax phantom.^26^ Oliver and Monajemi compared the calculated doses in a 2‐mm‐thick skin structure on a thorax phantom with those in the outermost 0.5‐mm‐thick shell of the skin using Monte Carlo simulation at a voxel size of 0.5 mm.^27^ When a bolus was present on the skin, the dose in the outer 0.5 mm agreed with that in the entire 2 mm structure, while without a bolus the mean dose in the 2 mm structure was by about 15%–20% higher than in the outer 0.5 mm. Therefore, the skin NTCPs calculated here using the Lyman model are overestimates of the NTCPs calculated at a depth of 0.07 mm and the overestimates without TTField arrays are higher than those with TTField arrays. Yet, even for large superficial glioblastoma target volumes using non coplanar beams with direct entrances, avoiding longer paths through brain tissue between entrance and target volume to reduce brain exposure the calculated NTCP values for skin ulcers, remained below 1.5% without TTField arrays and below 5.5% with TTField arrays fixed at a constant central position. The calculated NTCP enhancement factors may underestimate the true factors. However, the absolute NTCPs have to be considered as overestimates. Mitigation techniques to reduce the effect of the TTField arrays on the dose buildup, such as position variation of the TTField arrays and irradiation without arrays at the days of array change, may halve the increase in NTCP caused by fixed arrays. Skin‐NTCP calculation uncovers significant interpatient heterogeneity and may be used to stratify patients into high‐and low‐risk groups of skin toxicity.

## AUTHOR(S) CONTRIBUTION

Nika Guberina and Martin Stuschke were involved in study concepts, statistical analysis, and manuscript preparation.

Nika Guberina, Maja Guberina, Youness Nour, and Martin Stuschke were involved in study design.

All authors were involved in data acquisition, quality control of data and algorithms, and manuscript review.

Nika Guberina, Youness Nour, and Martin Stuschke were involved in data analysis and interpretation.

Nika Guberina, Christoph Pöttgen, Youness Nour, and Martin Stuschke were involved in manuscript editing.

## Supporting information


**Fig. S1** Depiction of the correlation graph of the accumulated min surface dose to the hottest 1 cm² [%] in the superficial scalp layer (2 mm and 4 mm shell contour): Highlighting correlation graph between Acuros XB and Monte Carlo Simulation (MC) implemented in Prosoma version 4.2. (based on the VMC++ and XVMC‐ code) without TTField electrodes (2 mm blue, 4 mm purple; Acuros and MC) and with TTField electrodes which are moved around their center (2 mm green, 4 mm red; eAcuros and eMC).
