# Supplementary material for: Dosimetric impact of the positioning variation of tumor treating field electrodes in the PriCoTTF‐phase I/II trial
Source: J Appl Clin Med Phys. 2021 Jan 3;22(1):242–50. doi: 10.1002/acm2.13144 (PMC7856507; doi:10.1002/acm2.13144)
Supplement: Supplementary file 1 — Fig. S1 Depiction of the correlation graph of the accumulated min surface dose to the hottest 1 cm² [%] in the superficial scalp layer (2 mm and 4 mm shell contour): Highlighting correlation graph between Acuros XB and Monte Carlo Simulation (MC) implemented in Prosoma version 4.2. (based on the VMC++ and XVMC‐ code) without TTField electrodes (2 mm blue, 4 mm purple; Acuros and MC) and with TTField electrodes which are moved around their center (2 mm green, 4 mm red; eAcuros and eMC). [file ACM2-22-242-s001.docx]

| **Suppl. Figure 1** | Depiction of the correlation graph of the accumulated min surface dose to the hottest 1 cm² [%] in the superficial scalp layer (2 mm and 4 mm shell contour): Highlighting correlation graph between Acuros XB and Monte Carlo Simulation (MC) implemented in Prosoma version 4.2. (based on the VMC++ and XVMC- code) without TTField electrodes (2 mm **blue,** 4 mm **purple**; Acuros and MC) and with TTField electrodes which are moved around their center (2 mm **green,** 4 mm **red**; eAcuros and eMC). |
| --- | --- |


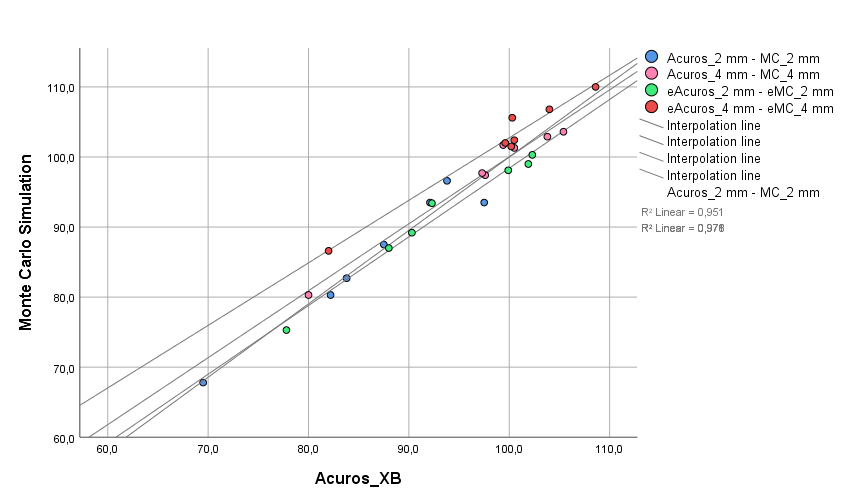


Accumulated min surface dose to the hottest 1 cm^2^ [%]

Accumulated min surface dose to the hottest 1 cm^2^ [%]
